# Neuroblastoma SH-SY5Y Cell Differentiation to Mature Neuron by AM580 Treatment

**DOI:** 10.1007/s11064-022-03730-w

**Published:** 2022-09-06

**Authors:** Aojie Cai, Zehong Lin, Nana Liu, Xiao Li, Jingmin Wang, Ye Wu, Kai Gao, Yuwu Jiang

**Affiliations:** 1 Department of Pediatrics, Peking University First Hospital, No. 1 Xi’an Men Street, West District, Beijing, 100034 China; 2 Beijing Key Laboratory of Molecular Diagnosis and Study on Pediatric Genetic Diseases, Beijing, China; 3 Children Epilepsy Center, Peking University First Hospital, Beijing, China; 4 Department of Neurology, Affiliated Children’s Hospital of Capital Institute of Pediatrics, Beijing, 100020 China; 5 Key Laboratory for Neuroscience, Ministry of Education/National Health and Family Planning Commission, Peking University, Beijing, China; 6 Center of Epilepsy, Beijing Institute for Brain Disorders, Beijing, China

**Keywords:** AM580, SH-SY5Y cell, Neuronal differentiation, Mature neuron, RNAseq

## Abstract

Neuroblastoma is a type of developmental childhood cancer that arises from the neural crest. It is the most common pediatric solid tumor in the world. AM580 is a powerful cyto-differentiating molecule on acute promyelocytic leukemia cells and induced pluripotent stem cells, but its effect on neuroblastoma is still unknown. In this study, the neuronal differentiation impact of AM580 was investigated using the human neuroblastoma cell line SH-SY5Y as a model. AM580 successfully stimulated the SH-SY5Y cells to develop into neuron-like cells. Functional enrichment analysis of RNAseq data revealed that differentially expressed genes (DEGs) were substantially enriched for GO keywords and KEGG pathways linked to neuron development. Some potassium ion channel genes associated with neuronal excitation, such as *KCNT1*, were shown to be upregulated. Through the MEA tests, we found the AM580-induced neurons possessed electrical spikes as mature neurons. AM580 also induced the neuronal marker β-tubulin III and mature neurons marker Neurofilament H. Our study proved that AM580 can promote the differentiation of neurons and has the potential to treat neuroblastoma, neurodevelopmental and neurodegenerative diseases.

**Supplementary Information:**

The online version contains supplementary material available at 10.1007/s11064-022-03730-w.

## Introduction

Neuroblastoma is still the most common pediatric solid tumor, accounting for ~ 15% of childhood cancer-related mortality [1]. Neuroblastomas have a wide range of clinical behavior, from spontaneous regression or differentiation into a benign ganglioneuroma, to relentless and refractory progression [2]. Neuroblastoma is a developmental tumor [3]. Promoting differentiation of cancer cells may be an effective candidate for treatment of neuroblastoma.

Neuron differentiation is a complex biological process regulated by multiple signaling pathways as TGF-β signal pathway [4], SHH signal pathway [5], Wnt signal pathway [6], Notch signal pathway [7] and RARs signal pathways [8]. Clarification of the mechanism of neuron differentiation is beneficial to cure the diseases of the neural system, such as neuroblastoma, neurodevelopmental and neurodegenerative diseases.

SH-SY5Y is a neuroblastoma cell line. All-trans retinoic acid (ATRA) can induce SH-SY5Y to develop neurons via activating nuclear retinoic acid receptors (RARs) [9, 10]. But ATRA is a fragile compound that is sensitive to light, oxidizes easily and becomes ineffective in aqueous solutions, and ATRA is the ligand of retinoic acid receptor (RAR) and retinoid X receptor (RXR) with a poor targeting. AM580 (CAS No.: 102121-60-8), also known as CD336, NSC608001 and Ro 40-6055, is a stable aminobenzoic acid that acts as a selective retinoic acid receptor alpha (RARα) agonist [11]. AM580 has powerful cyto-differentiating effect on acute promyelocytic leukemia cells [12, 13] and induced pluripotent stem cells [14, 15]. However, the impact of AM580 on neuroblastoma and neuronal development is still unknown. In this study, we found that AM580 can promote neuroblastoma SH-SY5Y differentiate to mature neurons.

## Methods

### Cell Culture of SH-SY5Y

The SH-SY5Y was kindly given by Professor Yun Wang of the Neuroscience Research Institute, Peking University. Cell cultures of SH-SY5Y were cultured in DMEM/F12 (Thermo Scientific, USA) with 10% (v/v) fetal bovine serum (FBS) (Gibco, USA) and 1% Penicillin–Streptomycin Solution (Gibco, USA). All cultures were incubated in a Thermo CO_2_ incubator at 37 °C with 95% air and 5% CO_2_ (v/v) and a humidity of 95%. The culture medium was changed twice a week. 70% ~ 80% confluent cultures used for passage to experiments.

### Drug Treatment

Unless illustrated in the paper, the SH-SY5Y was treated with different concentration of AM580 (Med Chem Express, USA) the next day after passage. As the stock AM580 solution (1 μM) is prepared with dimethyl sulfoxide (DMSO), the negative controls were control (non-treated) group and DMSO (treated with 1%DMSO) group.

### Time-Lapse Microphotography

To observe the dynamic changes of SH-SY5Y before and after AM580 treatment, the cytomorphology of SH-SY5Y was incubated in a INU microscope incubator (TOKAI HIT, Japan) at 37 °C with 5% CO_2_ (v/v) and recorded using an Olympus CKX41 inverted microscope. The videos recording of cell movement are played at 1800 times speed.

### Immunostaining and Western Blot

For immunostaining, cells were fixed with 4% Paraformaldehyde (PFA) for 20 min before being permeabilized with 0.3% Triton X-100 for 15 min. The cultures were then blocked with 3% bovine serum albumin (BSA) and 0.1% Triton X-100 in PBS for 1 h at room temperature and incubated with primary antibody rabbit anti-β-tubulin III (1:400, CST, USA) and mouse anti-neurofilament heavy polypeptide (1:400, CST, USA) at 4 °C overnight. After washing three times with PBS, the cultures were incubated with secondary antibody conjugated with Alexa Fluor 488 (1:500, Invitrogen) or Alexa Fluor 568 (1:500, Invitrogen, USA) for 1 h at room temperature. Nuclei were stained with 2 μg/mL Hoechst 33342 at room temperature for 15 min. Finally, the cultures were observed with a FV10i confocal microscope (Olympus, Japan). The 4th days cell proteins were abstracted with ice-cold RIPA lysis buffer (20 mM Tris–HCl (pH 8.1), 150 mM NaCl, 0.1% NP-40, 1% SDS, 0.5% sodium deoxycholate, 1 mM PMSF and 1 mM protease inhibitor cocktail) and western blot was performed to analyzed the expression of neuronal marker protein β-tubulin III (1:1000, CST) by iBlot-2 (25 V, 7 min, Thermo Scientific, USA). Blots were repeated three times for every set of experiments. Bands were normalized to GAPDH levels, and the density of the bands was measured using ImageJ analysis software (NIH, USA).

### Transcriptome Sequencing

Three samples were collected from Control group, DMSO group and 1 μM AM580-induced SH-SY5Y at 4 days, and mRNA was extracted for RNA-seq with TRIzol reagent (Life Technologies, USA). 2 µg RNA per sample was used as input material for the RNA sample preparations. Sequencing libraries were generated with the VAHTS mRNA-seq v2 Library Prep Kit for Illumina following the manufacturer’s recommendations. Index codes were added to attribute sequences for each sample. Then libraries were sequenced using an Illumina NovaSeq platform to generate 150 bp paired-end reads, according to the manufacturer’s instructions. Raw data (raw reads) of fastq format was firstly processed through primary quality control. In this step, clean data (clean reads) were obtained by eliminating read pairs that contain N more than 3 or the proportion of base with quality value below 5 is more than 20%, in any end, or adapter sequence was founded. The clean data of each sample was more than 6 GB. All downstream analyses were based on the clean data with high quality. The alignment of paired-end clean reads to the reference genome was with TopHat (v2.1.1). Differential expression analysis between two conditions was performed using Cufflinks (v2.2.1). Differentially expressed genes (DEGs) were defined as those for which the P-value is below 0.01 and the absolute value of log2 Fold Change (|log2FC|) is not less than 1.

### Functional Enrichment Analysis

GO and KEGG enrichment analysis of DEGs sets were implemented by KOBAS 3.0 [16]. GO terms and KEGG pathways with adjusted P-value below 0.05 were considered as significantly enriched by differential expressed genes.

### Real-Time PCR

Total RNA was extracted with TRIzol and checked using a Nanodrop 2000 (Agilent Technologies, Santa Clara, CA, USA) in different concentration dose of Am580 at 4th days. Quantitative PCR was carried out with BeyoFast™ SYBR Green qPCR Mix (2 ×) (Beyotime, China) in a 20 μL final volume according to the manufacturer’s instructions. Samples were run for 40 cycles at default thermal cycling conditions for SYBR Real-Time PCR (stage 1: 1 cycle, 95 °C for 2 min; stage 2: 40 cycles, 95 °C for 15 s, 60 °C for 30 s, 95 °C for 15 s). The primer sequences were as follows: *GAPDH* forward, 5′-GTGGACCTGACCTGCCGTCT-3′; *GAPDH *reverse, 5′-GGAGGAGTGGGTGTCGCTGT-3′; *KCNT1* forward, 5′-CCGGACCTTCGAGTTTGACG-3′; *KCNT1* reverse, 5′-GTCGCTCATCTTGAAGCCG-3′. Each sample was performed routinely in triplicate. The relative expression levels were analyzed using the 2^-ΔΔCT^ method.

### Microelectrode Arrays (MEA) Recording

Electrical signals of AM580-induced SH-SY5Y have been acquired by a high-density MEA (3Brain, Switzerland). This device was able to record spontaneous firing of AM580-induced SH-SY5Y. SH-SY5Y was cultured and induced by 1 μM AM580 on MEA CMOS chips. The spikes of each culture at 4 days after AM580-treatment were monitored for up to 5 min and then detected off-line with analysis software BrainWave 4 (3Brain, Switzerland).

### Data Statistics

Data are calculated as the mean ± standard error of the mean (SEM). The Ordinary One-way ANOVA is used to analyzed the between‐group differences. P value < 0.05 was considered statistically significant.

## Results

### AM580 induced SH-SY5Y to neuron-shape cells

**Fig. 1:**
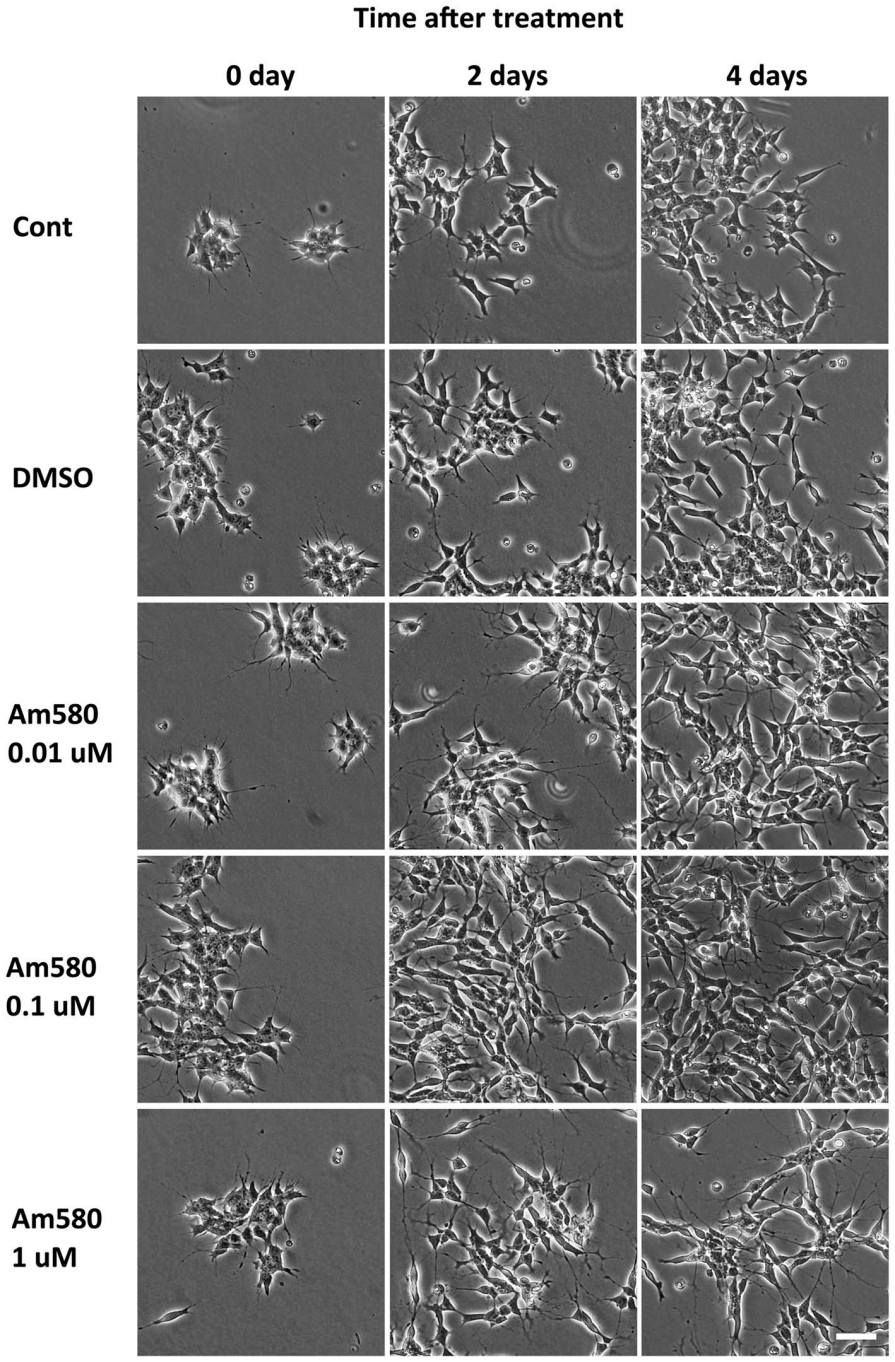
Microscopic observation of SH-SY5Y after AM580 treatment with different concentrations. Bar = 50 μm

In order to study the impact of AM580 on SH-SY5Y, we first observed the effect of AM580 on cell morphology. We treated the cell line with different concentrations of AM580, including 0.1 μM, 1 μM, 10 μM, and took phase contrast pictures on 0 day, 1 day, 2 days and 4 days. We tracked the same area of cell cultures for 4 days after AM580 treatment (Fig. 1). Normal SH-SY5Y has a star shape with several short cell processes. 0.1% DMSO treated SH-SY5Y cell had no difference with normal SH-SY5Y morphology. After 0.01 μM AM580 treatment, SH-SY5Y cells were induced to form longer projections. And these projections always produced several branches. With the increase of AM580 concentration and the extension of time, AM580 promoted SH-SY5Y cells to connect with each other through long processes, forming a similar structure of neural network.

In order to observe the dynamic changes of SH-SY5Y after AM580 treatment, the morphological changes of SH-SY5Y before and after the treatment of AM580 were observed by time-lapse microphotography. We recorded a same view of SH-SY5Y cell culture before (for 0 day) and after 1 μM AM580 treatment (for 4 days). We found the cell process elongation of SH-SY5Y cells was more obvious and always did not retract quickly as normal after AM580 treatment (Supplement Videos 1 and 2). Sometimes the cells even produced a few bent cell process (the axon-like process extend in the other direction after being anchored at one point) or branches. In addition, we also observed some cell division, indicating that AM580 could not, at least not completely, inhibit cell division.

### Terms and Pathways Associated with Neuron Differentiation were Enriched by RNAseq and Differentially Expressed Genes (DEGs) Analysis

We performed RNAseq to analyze the effect of AM580 on the transcriptional profile of SH-SY5Y. Genes with relative expression levels that showed |log2 FC| ≥ 1 and p ≤ 0.01 were considered as DEGs. Of the 1232 DEGs detected after 1 μM AM580 for 4 days, 818 up-regulated genes and 414 down-regulated genes.

In order to determine the biological function of DEGs after AM580 treatment in the SH-SY5Y cell line, the 1232 DEGs were submitted to KOBAS 3.0 for GO and KEGG enrichment analysis. The results revealed enrichment of 306 GO terms and 56 KEGG pathways (Corrected P-Value ≤ 0.01).

**Fig. 2:**
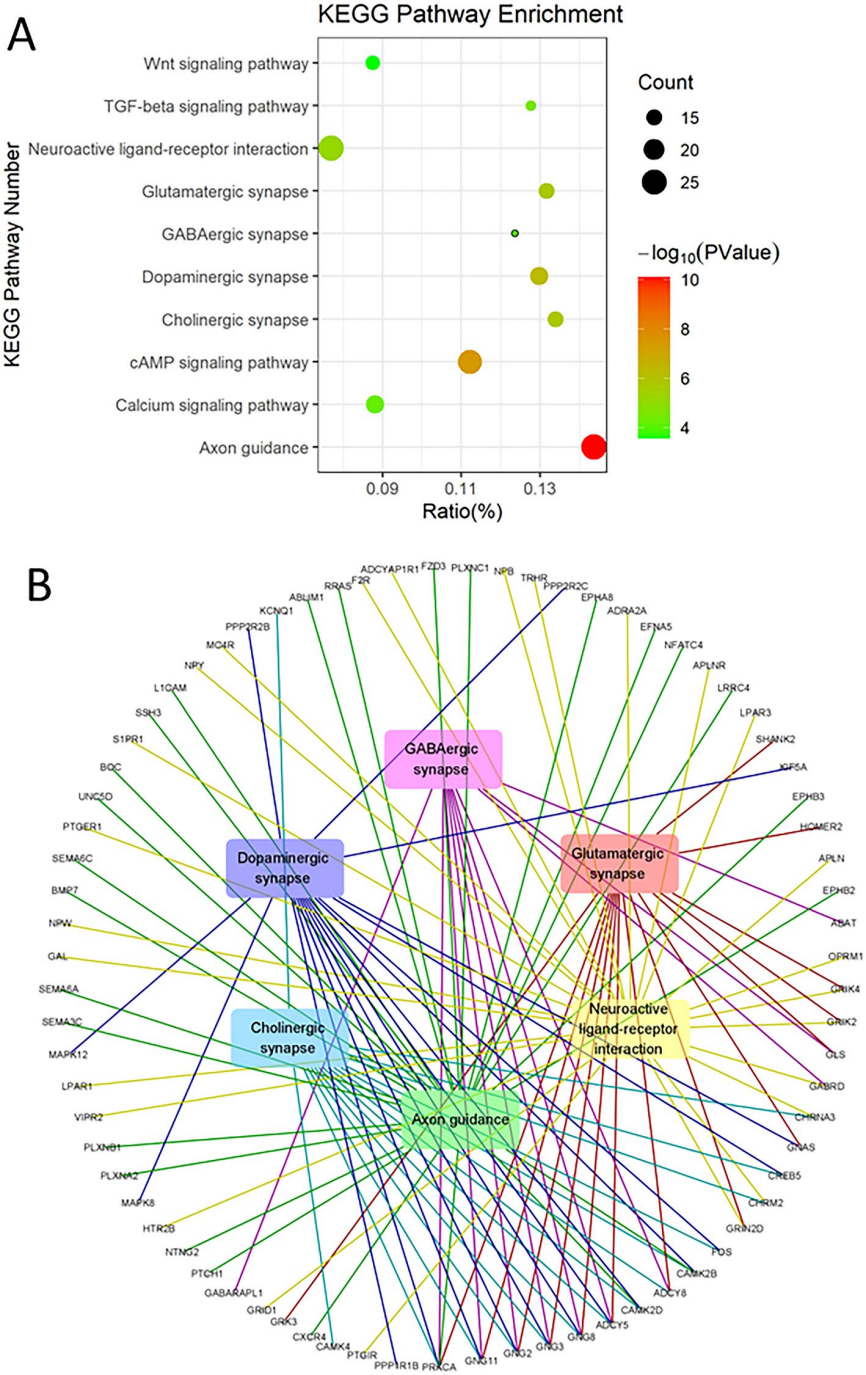
KEGG pathway enrichment of DEGs. **A** Bubble plot shows the functional enrichment of 10 neuron related KEGG pathways. The graph size of bubbles represents the number of enriched DEGs in each pathway, the color of the bubbles represents the P value, the vertical axis represents the name of the relevant KEGG pathway, and the horizontal axis represents the percentage of DEGs in all genes of each pathway. **B** The interactions of significantly enriched KEGG pathways with DEGs

The top 10 neurobiology-related significant enrichments of KEGG pathways are shown in Fig. 2A. Many neuronal differentiation-related pathways were enriched, including Wnt signaling pathway, TGF-beta signaling pathway, neuroactive ligand-receptor interaction, glutamatergic synapse, GABAergic synapse, dopaminergic synapse, cholinergic synapse, cAMP signaling pathway, calcium signaling pathway and axon guidance. Then, we constructed a network of neuron-related pathways (neuroactive ligand-receptor interaction, glutamatergic synapse, GABAergic synapse, dopaminergic synapse, cholinergic synapse, and axon guidance) with DEGs (Fig. 2B). We found that *PRKCA*, *GNG11*, *GNG2*, *GNG3*, *GNG8*, *ADCY5*, *CAMK2D* and *ADCY8* are linked to more than 3 pathways, indicating that those genes are important in AM580-induced SH-SY5Y differentiation.

**Fig. 3:**
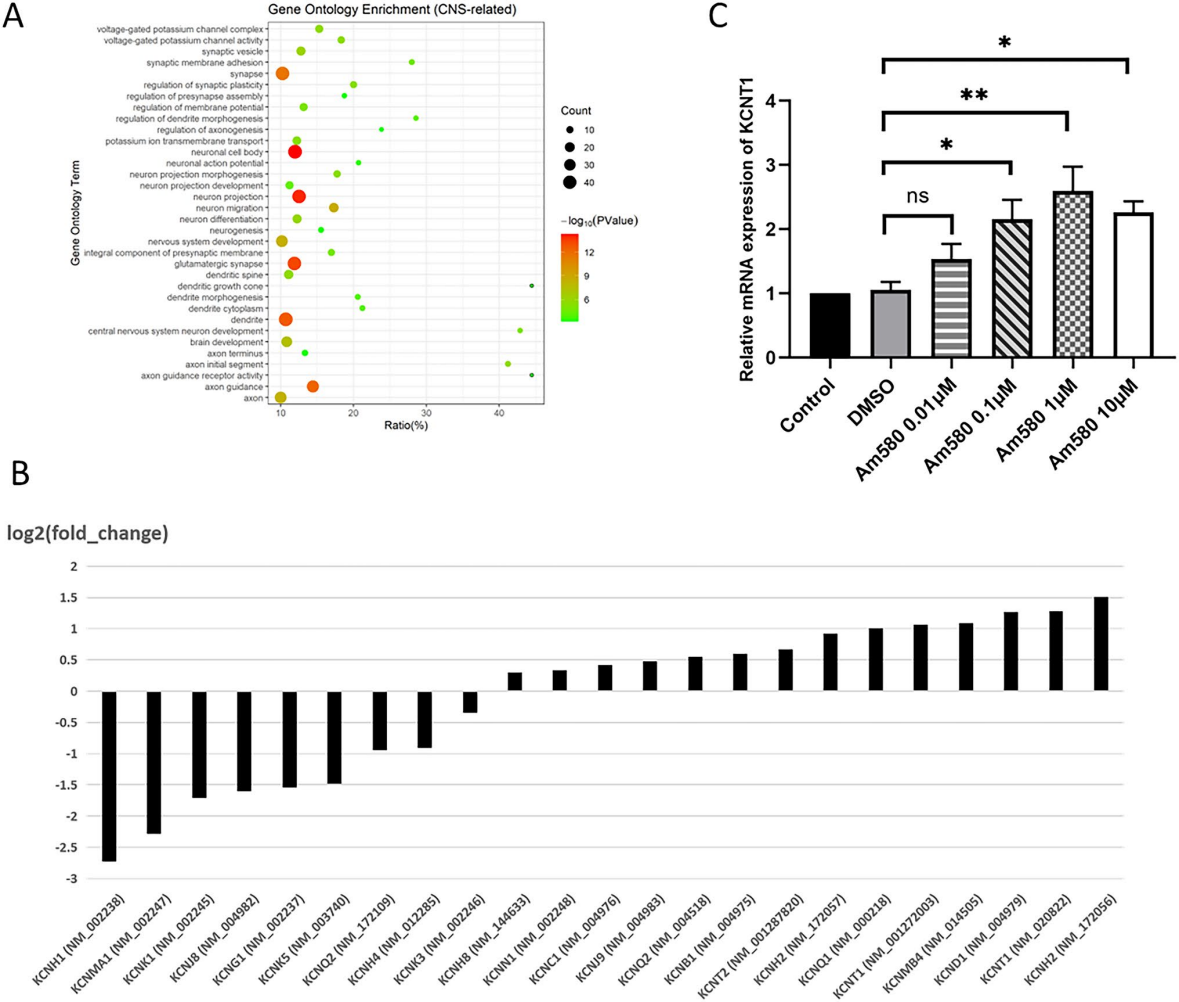
GO term enrichment of DEGs. **A** Bubble plot shows the functional enrichment of 34 neuron-related GO terms. The graph size of bubbles represents the number of enriched DEGs in each pathway, the color of the bubbles represents the P value, the vertical axis represents the name of the relevant GO terms, and the horizontal axis represents the percentage of DEGs in all genes of each pathway. **B** Bar plots showing the expression change of normal SH-SY5Y with AM580 treated SH-SY5Y for 4 days. The vertical axis represents the number of log2 FC of the expression change, and the horizontal axis represents the KCNs genes (transcripts). **C** The mRNA expression of *KCNT1* in the different dose of Am580-treated and nontreated groups at 4 days (n = 4). **P value < 0.01, *P value < 0.05

Thirty-four neuron-related significant enrichments of GO terms are shown in Fig. 3A. We found that synapse, neuronal cell body, neuron projection, glutamatergic synapse, dendrite cytoplasm and axon guidance were with high count and the most significant P value. Dendritic growth cone, central nervous system neuron development, axon initial segment and axon guidance receptor activity were with high ratio of DEGs in these GO terms. All those findings indicate that AM580 can induce and promote neuron development.

**Fig. 4:**
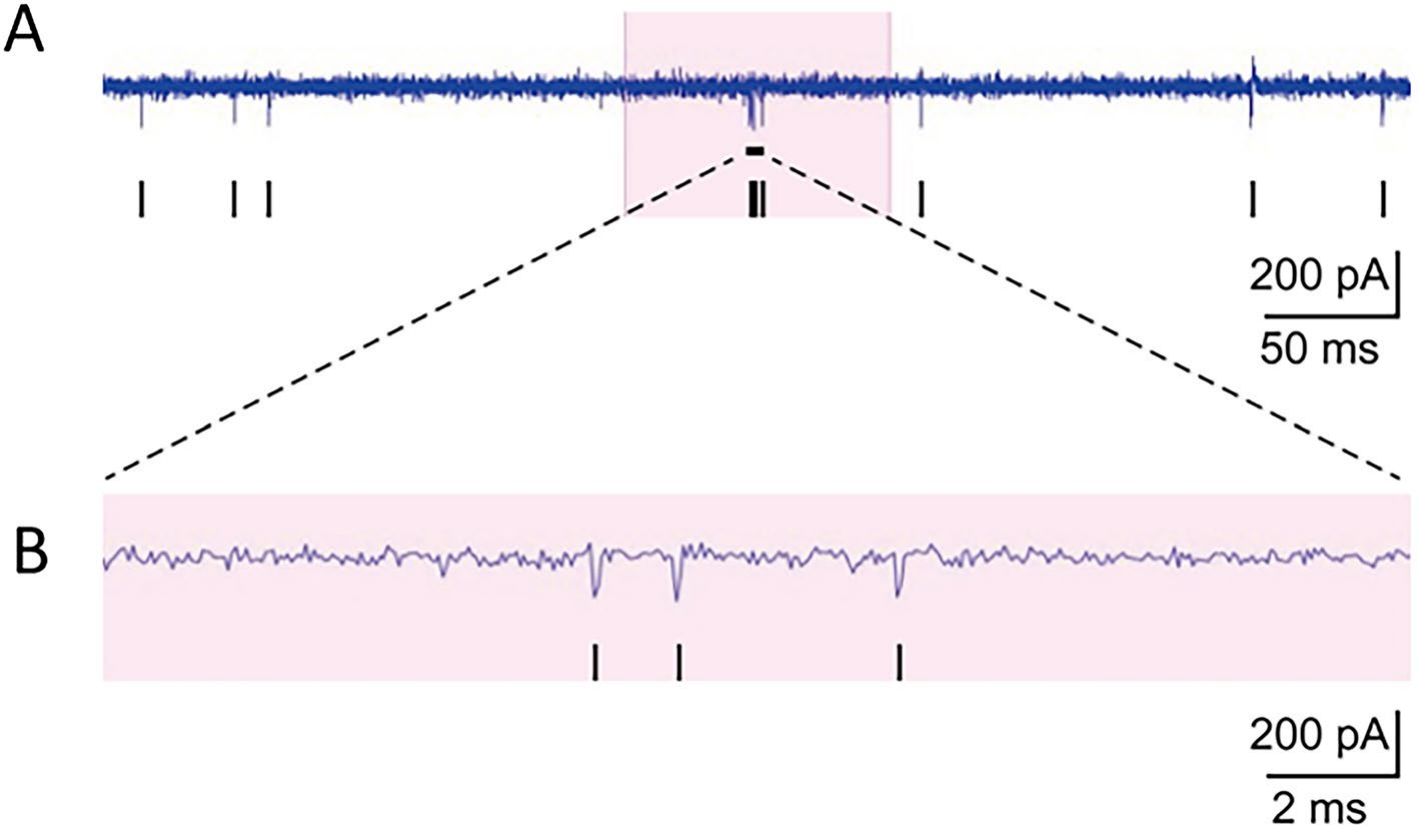
MEA assay of AM580-induced neuron. **A** The record of field potential change in about 0.5 s. **B** A zoom-in image of marked area in (**A**)

Potassium channels are the most numerous and widespread ion channels in the human body and are associated with a series of neurological processes, including neuron excitation. We also found the related GO terms as voltage-gated potassium channel complex, voltage-gated potassium channel activity and potassium ion transmembrane transport, were enriched (Fig. 4A). We analyzed the transcriptional profile of KCNs in the RNAseq data (Fig. 3B). We found that some KCN genes were significantly upregulated (log2 FC ≥ 1) as *KCNT1*, *KCNMB4*, *KCND1* and *KCNH2*. And some KCN genes were significantly downregulated (log2 FC ≤ − 1), including *KCNH1*, *KCNMA1*, *KCNK1*, *KCNJ8*, *KCNG1* and *KCNK5*. The mRNA expression of the neuronal ion channel gene *KCNT1* also be checked with qPCR at different concentration dose of Am580-treated for 4 days, and We found that only 0.1um of Am580 was needed to significantly increase the expression of *KCNT1* and can induce neuronal differentiation (Fig. 3C). The change of KCN expression indicates the change of cellular excitation.

### AM580 Promoted SH-SY5Y to Functional Neuron

In order to examine the electroactivity of AM580-induced neuron-sharp SH-SY5Y cell, we perform the MEA experiment. Our results, which acquired from AM580-induced SH-SY5Y cell cultivated on the surface of MEA chips, showed that the spontaneous spiking activity exists in AM580-induced SH-SY5Y (Fig. 4), while not in the untreated SH-SY5Y or DMSO-treated SH-SY5Y (data not shown). These results indicated that AM580 promotes SH-SY5Y cells to functional neurons.

### β-Tubulin III and NFH were Distributed in Axon-Like Processes of AM580-Induced SH-SY5Y

**Fig. 5:**
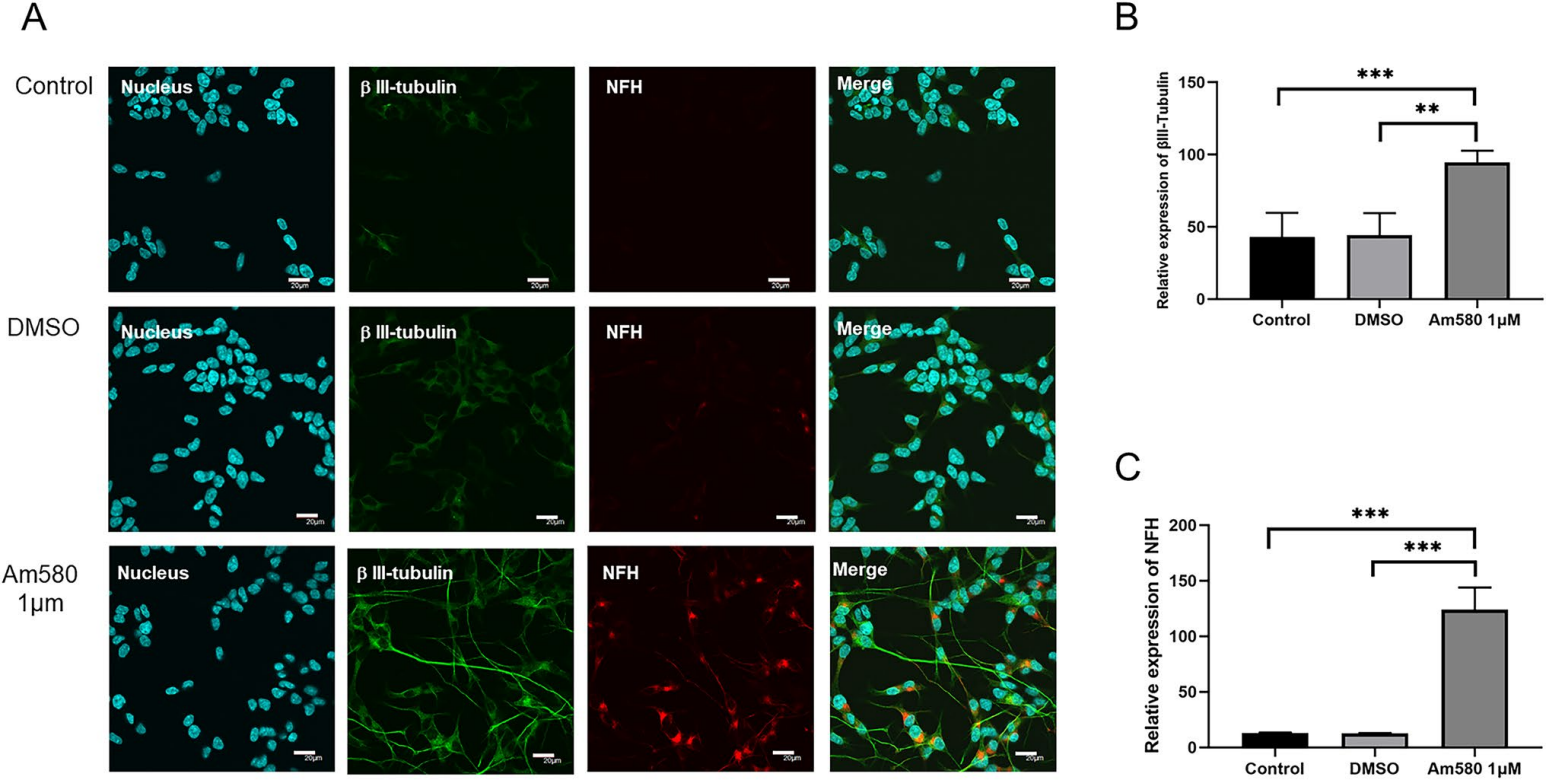
Immunostaining of 1 μM AM580-treated, DMSO-treated SH-SY5Y and control group. **A** βIII-tubulin was used to stain neurons (green), NFH (neurofilament-H) was used to stain mature neurons (red), Hoechst 33258 was used to stain nuclei (blue) and overlapping layers of fluorescence channels. Scale bar = 20 μm. **B**, **C** The relative expression of βIII-tubulin (**B**) and NFH (**C**) in the AC-treated (n = 3), DMSO (n = 6) and control groups (n = 6) at 4 days. **P value < 0.01, ***P value < 0.001

In order to determine the development of SH-SY5Y cells induced by AM580 into neurons, we observed the neuron-specific markers β-tubulin III and mature neurons marker neurofilament heavy polypeptide (NFH) in cells by immunofluorescence. Compared with the control group, we found that β-tubulin III (Fig. 5A and B, Fig. S1) and NFH were significantly distributed in elongated cellular processes of AM580-treated SH-SY5Y as axons in neurons (Fig. 5A and C), suggesting that these processes are nerve fibers and neurons may develop into mature neurons.

## Discussion

AM580 is a stable retinoic acid benzoic acid derivative and selective RAR-α agonist that significantly reduces Th1 cytokine production and has anti-inflammatory and neuroprotective effects [17]. The RAR signal pathway is essential in neural development. In 1933, Hale F found that Vitamin A deficient diet resulted in “Pigs born without eyeballs” [18]. In the 1980s, RA was found to have neural differentiation activity and induce neuroblastoma and embryonal carcinoma cells to neurons [19, 20]. RARs are a family RA receptor, including RARα, RARβ, RARγ. RARα is a critical receptor in neural development, including neuron differentiation [21] and synaptic plasticity [22]. In this study, we first found that the selective agonist AM580 can induce the SH-SY5Y differentiation, suggesting the activation of RARα is enough to promote neuroblast to neuron (at least in neuroblastoma cell line SH-SY5Y).

When treated with 1 μM AM580 for 4 days, the neuroblastoma cell line SH-SY5Y was promoted to be neuron-shape cell with expressing the neuron specific cytoskeleton protein beta-tubulin III and neurofilament-H. And we also found AM580 can induce the expression of ion channel genes, as *KCNT1* and *KCNH2* and promote the SH-SY5Y cell to functional excitatory neuron. Neurofilaments (NFs) are a neuron-specific class of intermediate filaments (IFs). Neurofilaments are heteropolymers composed of four subunits, including NF-L (neurofilament light polypeptide), NF-M (neurofilament middle polypeptide), NF-H (neurofilament heavy polypeptide), and α-internexin in central nervous system [23]. During neurons mature, the expression of nestin and vimentin replaced by NF subunits, which might consolidate axon elongated process and neural network connection [24, 25], so NFs were always regarded as markers of mature neurons [25]. The study of NFs knockout mice showed that loss-of-function of NFs resulted the loss of axons, supporting the role of NFs in axon growth and stabilization [26]. NF-H and NF-M subunits stabilized axon by blocking turnover of the NF network [27]. In our study, we found NF-H distributed in axon of AM580-induced SH-SY5Y, indicating differentiated SH-SY5Y cells were mature neuron and might take role in the outgrowth and elongation of AM580-induced axon, and stabilized it not to retract easily (Supplement Video).

Potassium channels are the indispensable components in neuronal electric activity. The potassium channel ERG1, encoded by *KCNH2*, is expressed in a series of cells including neurons, smooth muscular cells, and cardiac cells, even cancer cells [28]. Mutation of *KCNH2* would result in epilepsy, indicating its role in neuronal electrophysiology [29]. Slo2.2, encoded by *KCNT1*, is a potassium channel in the high-conductance potassium channel family. It plays an important role in afterhyperpolarization and maintaining the resting potential and controlling the basal excitability of neurons [30]. Gain of function mutations of *KCNT1* induce epilepsy [31], while loss of function of *KCNT1* results in autism [32]. In this study, we found that AM580 induced the expression of ion channel genes of neurons, as *KCNT1*, promote the SH-SY5Y cell to functional excitatory neuron. Our data indicated that RAR signal pathway involved in the start of neuronal differentiation by upregulating neuronal ion channels, as *KCNT1*, which can be used to explain partly why AM580 induced the SH-SY5Y cells to a functional neuron with electroactivity.

In terms of application progress, Am580, a RARα-specific agonist, has been reported relatively rarely in previous studies, mainly focusing on regulating the microenvironment of cell proliferation. Initial studies reported that it exhibited anti-angiogenic activity in vivo, might have potential therapeutic efficacy in various angiogenesis-dependent disorders [33]. Am580 inhibited the wnt pathway and have anticancer effect [34], studies have proved that Am580 could inhibits endometrial cancer cell growth [35]. In the nervous system and cyto-differentiation, studies have reported that Am580 could powerful and selective promoted granulocytic differentiation of acute promyelocytic leukemia cells [12]. It also can inhibit microglial activation, shifting them to a homeostatic and neuroprotective phenotype, thus playing a beneficial role in the treatment of Alzheimer's disease [36]. In addition, AM580 protects retinal cells from diabetes-induced apoptosis by inducing neurotrophic factors [17] Neuroblastoma-derived SH-SY5Y cells have become an excellent model for the study of AD and neural study. On the basis of our results, we conclude that AM580 promotes the differentiation of neuroblastoma SH-SY5Y. AM580 is a selective RARα agonist. Our study provides direct evidence on the important effect of RARα signaling in neuron differentiation. Although more work needs to be done later, we established a differentiated neurons that have a close resemblance to human neurons and provided that AM580 is indicated to be a potential therapeutic agent in neurodegenerative disorders and neurodevelopmental diseases. The conclusion was based on the observation of neuroblastoma cell line SH-SY5Y in vitro. It might not reflect processes in vivo. we will confirm our results in mice in further study.

## Supplementary Information

Below is the link to the electronic supplementary material.

Supplementary file1 (TIF 3138 KB)

Supplementary file2 (MP4 36624 KB)

Supplementary file3 (MP4 143006 KB)

## Data Availability

The original data presented in the study are included in the article materials, further inquiries can be directed to the author/corresponding authors.

## Abbreviations

DEGs: Differentially expressed genes
ATRA: All-trans retinoic acid
RARs: Retinoic acid receptors
RARα: Retinoic acid receptor alpha
NFH: Neurofilament heavy
log2 FC: log2 Fold change
NFs: Neurofilaments
IFs: Intermediate filaments
NF-L: Neurofilament light
NF-M: Neurofilament middle
NF-H: Neurofilament heavy
FBS: Fetal bovine serum
BSA: Bovine serum albumin
MEA: Microelectrode arrays
