# Supplementary figures and images for: Neuroblastoma SH-SY5Y Cell Differentiation to Mature Neuron by AM580 Treatment
Source: Neurochem Res. 2022 Sep 6;47(12):3723–32. doi: 10.1007/s11064-022-03730-w (PMC9718880; doi:10.1007/s11064-022-03730-w)

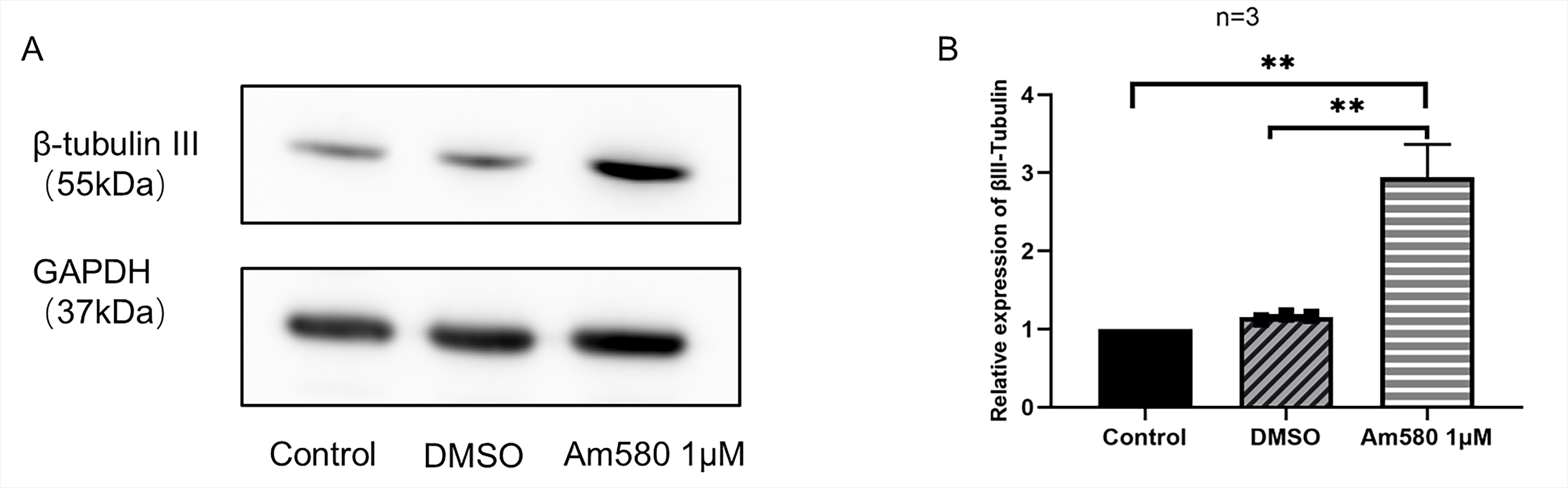

Supplement: Supplementary file 1 — Supplementary file1 (TIF 3138 KB) [file 11064_2022_3730_MOESM1_ESM.tif]
